# Current Understanding on the Heterogenous Expression of Plastic Depolymerising Enzymes in *Pichia pastoris*

**DOI:** 10.3390/bioengineering12010068

**Published:** 2025-01-14

**Authors:** Shuyan Wu, David Hooks, Gale Brightwell

**Affiliations:** 1 AgResearch Ltd., Grasslands, Palmerston North 4442, New Zealand; david.hooks@agresearch.co.nz (D.H.); gale.brightwell@agresearch.co.nz (G.B.); 2 New Zealand Food Safety Science and Research Centre, Tennent Drive, Massey University, Palmerston North 4474, New Zealand

**Keywords:** biodegradation, depolymerisation, polymer-degrading enzyme, heterologous expression, *Pichia pastoris*

## Abstract

Enzymatic depolymerisation is increasingly recognised as a reliable and environmentally friendly method. The development of this technology hinges on the availability of high-quality enzymes and associated bioreaction systems for upscaling biodegradation. Microbial heterologous expression systems have been studied for meeting this demand. Among these systems, the *Pichia pastoris* expression system has emerged as a widely used platform for producing secreted heterologous proteins. This article provides an overview of studies involving the recombinant expression of polymer-degrading enzymes using the *P. pastoris* expression system. Research on *P. pastoris* expression of interested enzymes with depolymerising ability, including cutinase, lipase, and laccase, are highlighted in the review. The key factors influencing the heterologous expression of polymer-degrading enzymes in *P. pastoris* are discussed, shedding light on the challenges and opportunities in the development of depolymerising biocatalysts through the *P. pastoris* expression system.

## 1. Introduction

The escalating plastic crisis has entered a more intricate phase with the introduction of new biodegradable polymers and plant-based alternatives. Despite efforts to minimise plastic waste, the growth of plastic products continues unabated. Many plastics face impending phase-outs due to recycling challenges and adverse environmental and health impacts, such as microplastic pollution. A rising dominance of biodegradable plastics in the market is now offering a potential solution. However, the green claims surrounding bioplastics are met with scrutiny, as they disrupt current plastic-recycling streams and, at times, fail to readily degrade in natural environments [1]. Consequently, plastic biodegradation in nature is at low efficiency. The engineered enzymatic breakdown of these materials could be a promising approach for sustainable waste management and bioplastic recycling. Therefore, scientists are actively researching and developing “artificial enzymes” (often called “plastic-eating enzymes”) to significantly speed up the breakdown of plastic waste, allowing for a more efficient recycling and environmental cleanup process.

Therefore, active research activities have isolated polymer-degrading enzymes derived from diverse microbial species. Predominantly classified as hydrolases and oxidoreductases, these enzymes play pivotal roles in the breakdown of plant polymers. Noteworthy examples encompass laccases (EC 1.10.3.2), cutinases (EC 3.1.1.74), esterases (EC 3.1.1.1), lipases (EC 3.1.1.3), and manganese peroxidases (EC 1.11.1.13) [2]. Particular enzymes, including PET hydrolases, cutinases, and polyesterases, are being explored for enhancing the depolymerising ability by enzymatic engineering [3]. These enzymes exhibit significant structural diversity and varied catalytic capabilities, demonstrating specific activities towards different polymers.

However, the microbial degradation rate remains sluggish under constrained environmental conditions. The development of these enzymes focuses on enhancing their catalytic activity, substrate specificity, and stability across a range of environmental conditions. Recent advancements have included directed evolution and protein-engineering techniques to create more robust depolymerases. Currently, the technology is used to make enzymes more tolerant under varying conditions and more flexible to be engineered [4].

To date, *Escherichia coli* has emerged as a widely used expression system for the heterologous production of depolymerases, primarily derived from microbial sources such as bacteria and fungi [1]. This expression system has demonstrated tangible degradation capabilities on polyethylene terephthalate (PET) [5]. However, it is imperative to acknowledge the limitations of *E. coli*, as it is not universally adept at expressing active enzymes. *E. coli* frequently engages in erroneous post-translational processes for proteins containing cysteine residues [6,7], and encounters challenges in forming disulfide bonds [8,9]. Additionally, eukaryotic enzymes expressed in *E. coli* exhibited a short half time [10] or quickly lost catalytic activity [11,12].

To overcome the limitations of *E. coli* expression systems, yeasts have been used to achieve high-yield protein overexpression [13]. Yeast expression systems have been adapted to produce varied polymer-degrading enzymes, using yeast hosts such as *Cryptococcus* sp. [14,15], *Saccharomyces cerevisiae* [16], and *Pichia pastoris* (*P. pastoris*). The performance of heterogeneous expression will be influenced by the choice of different expressing host cells [17,18,19], suggesting that using a host with similar genetic characteristics will improve the heterogenous protein expression.

Among them, *P. pastoris* (now *Komagataella phaffii*) is a well-recognised expression system for producing heterologous proteins [13]. *Pichia pastoris* is an attractive host for expressing these enzymes due to its ability to secrete active proteins efficiently, making it a promising platform for producing polymer-degrading enzymes at scale. Current evidence suggests that recombinant expression in *P. pastoris* could produce enzymes carrying more stabilised structures and activity [20]. For example, the expression of a fungal cutinase, demonstrating 90% catalytic activity over a span of 48 h at 50 °C, was reported by Kazenwadel, Eiben et al. [21]. Additionally, a bacterial laccase has been expressed and remains active for a duration of 10 days at 30 °C, as documented by Lu, Wang et al. [22]. Furthermore, a fungal lipase has been expressed that exhibits detectable activity for up to 10 days, as described by Jallouli et al. [23]. Notably, the utilisation of high-cell-density fermentation has facilitated enzyme production with low maintenance demand and minimised downstream processing requirements, as outlined in the work of Abdulrachman, Thongkred et al. [24]. By fine-tuning these enzymes to work efficiently at ambient or composting temperatures, researchers aim to make bioplastic degradation more practical and cost viable. This enzymatic approach not only provides an environmentally friendly alternative to chemical recycling but also opens opportunities for circular economy strategies, where bioplastic waste is transformed into valuable monomers for new material synthesis.

*Pichia pastoris* has a proven track record of efficiently producing abundant yields of recombinant proteins [20]. In the context of expressing plastic depolymerising enzymes, *P. pastoris* is considered a good host for expressing a number of enzymes, as described below.

This article focuses on studies describing recombinant expression systems of microbial depolymerising-like enzymes (e.g., lipase, cutinase, and laccase) in *P. pastoris* over the last ten years. The review summarises key research, discusses the main factors affecting the heterogenous expression of polymer-degrading enzyme in *P. pastoris*, and postulates the following challenge of depolymerising enzyme production using *P. pastoris* expression systems.

## 2. Microbial Depolymerising Enzymes Commonly Expressed in *P. pastoris*

Firstly, *Pichia pastoris* is an ideal host for expressing heterologous lipase (E.C. 3.1.1.3) due to its lack of endogenous lipolytic activity when carrying an empty vector [25]. Lipases usually prefer water-insoluble acyl esters and emulsified substrates with long-chain acyl groups (≥C10) [26]. The potential activity of lipase on degrading biodegradable plastic polymers has been reported. A lipase from *Aspergillus niger* maintained its efficacy through recombinant expression in *P. pastoris*, and the recombinant lipase could degrade PLA 5000 (polylactic acid) (up to 87%), PLA 10,000 (up to 84%), and PCL 10,000 (polycaprolactone) (up to 78%) in a 72 h treatment [27]. A lipase B from a *Candida Antarctica* variant was reported to degrade PCL, and the ability was improved using *P. pastoris* as host for the lipase production [17,18].

Esterase hydrolyses the ester bonds of water-soluble acyl esters and emulsified glycerolesters with short-chain acyl groups (≤C8) [28]. Enzyme engineering has been used to improve esterase activity for hydrolysing long-chain fatty acids (C10–14) [29]. The group of enzymes often contains a pentapeptide motif (GYSLG) and the catalytic triad (Ser-Asp-His) as a shared feature of proteins in the esterase/lipase superfamily [28]. *P. pastoris* has been used for recombinantly expressing esterase (EC 3.1.1.1) for degrading plant polysaccharides [30]. A polyhydroxyalkanote depolymerase from a *Thermobifida* sp. isolate was expressed in *P. pastoris* with a C-terminal His6-tagged fusion and performed esterase-like activity of degrading bioplastic polymer poly-[(R)-3-hydroxybutyrate] (PHB) films with a measurable rate (870 ng/cm^2^) [31]. Since the *P. pastoris* genome contains non-specific esterase genes, the enzymatic activity of exogenous esterase is often evaluated with a reference group expressing an empty vector with inactive inserts [32]. Somehow, the activity of endogenous/non-specific esterase could be insignificant [33] or lost after the ultrafiltration process [34].

Cutinases are versatile enzymes within the esterase family, known for their ability to break down cutin, a natural polymer found in plant cuticles. Their capability extends beyond natural substrates, as they can hydrolyse synthetic polyesters like PET and various bioplastics. Cutinases (E.C. 3.1.1.74), as a class of serine esterase, can degrade high-molecular-weight polyesters (up to C18) and perform esterification/transesterification reactions, similar to lipase [35]. Thus, cutinases are often compared with lipases, which usually require interfacial activation to yield similar enzymatic activity [36]. Assessments of the polymer degradation efficiencies between cutinase and lipase [37,38,39] suggested that cutinase performs more promising depolymerisation against polyester. Cutinases are particularly effective in degrading hydrophobic polymers due to their amphipathic nature, which enables them to interact with and hydrolyse the ester bonds on the surface of plastics. The broad substrate specificity of cutinases makes them suitable for a wide range of applications in plastic waste degradation.

Cutinases, which are overexpressed in *P. pastoris*, are promising depolymerisers on breaking down synthetic polymers [40]. Exceptional candidates are reported, such as a mutant of cutinase from *Thermobifida cellulosilytica* capable of hydrolysing poly (butylene succinate) (PBS), with up to 92% weight loss within 96 h [41]; a cutinase from *Fusarium solani* that could completely degrade PBS film in 6 h [42]; a cutinase from *Aspergillous fumigatus* that completely degraded PCL and synthesised molecules with a molecular weight of 25,000 into dimers or monomers in 6 h [43]; and a glycosylated cutinase that originated from leaf and branch compost, causing 95% weight loss in amorphous PET film within 48 h [44]. In industrial settings, enzyme performance at elevated temperatures is often required to match the thermomechanical properties of plastics, and cutinases have been engineered to function optimally at these conditions. Thermostable cutinases have been developed that maintain high activity and structural integrity at temperatures exceeding 60 °C, making them valuable for large-scale bioremediation and recycling operations. These engineered enzymes have shown promise not only in plastic degradation but also in improving the recyclability of mixed and multilayered plastic materials.

Laccase (EC 1.10.3.2) belongs to the multicopper oxidase (MCO) family and has been found both in fungi and bacteria. Many bacterial laccases have been recombinantly expressed in *E. coli*, but the intracellular production led to a difficult enzyme purification [45]. Alternatively, heterogenous expression of bacterial laccase was conducted in *P. pastoris* to generate a higher yield with purified and active forms [22]. Fungal laccases tend to display redox activity with a higher enzyme yield than bacterial laccases, and many of them are modified (e.g., dye decolourisation in the textile industry) at a high temperature, high salt concentration, or extremely acidic or alkaline pH [46,47] to become adaptive in industrial applications for removing dye or toxic compounds. *P. pastoris* expressing laccase was reported to degrade micropollutants such as endocrine-disrupting chemicals and non-steroidal anti-inflammatory drugs [48]. High-expression yield of laccases in *P. pastoris* has been seen [48,49], as well as low yield [19]. It is assumed that the similar genetic character (e.g., shared codons and close GC content) between the expression host and the mother microorganism (carrying the target enzyme) could determine the efficiency of heterogenous expression [19]. Since laccases have roles in lignin degradation and wood modification [50], their ability to play a role in plastic depolymerisation is expected [51,52,53,54,55]. But the ability to degrade synthetic plastic polymers remains to be clarified.

## 3. Intrinsic Features of Enzyme Associated with the Functional Overexpression

**Figure 1:**
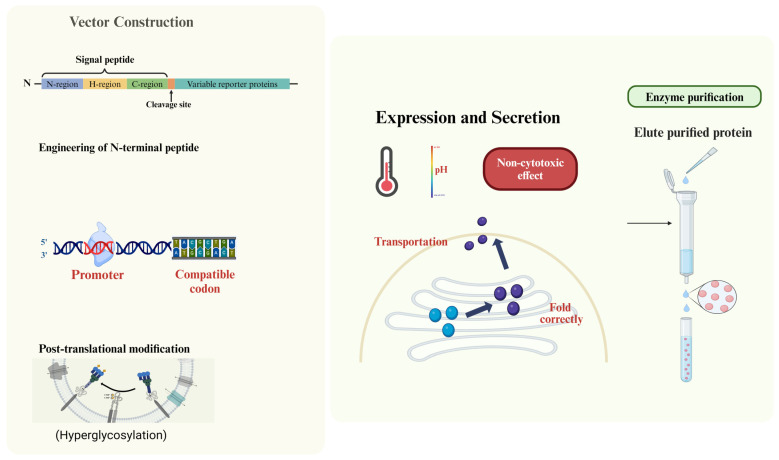
Highlighted factors influencing the effective functional overexpression of plastic-depolymerising enzymes in *P. pastoris*.

The effective functional overexpression of plastic-depolymerising enzymes in yeast is contingent upon several intrinsic and extrinsic features [25] (Figure 1). Enzymes must demonstrate stability within the conditions of yeast growth, encompassing the temperature and pH range conducive to yeast fermentation. Proper folding and maintenance of the correct conformation are imperative for functional activity, as misfolded proteins can result in diminished enzyme activity or degradation [19].

Resistance to degradation by yeast proteases is essential to maintaining stability and functionality during both expression and secretion. Efficient gene expression relies on compatibility with yeast promoters [23], with certain promoters, such as those inducible by specific carbon sources like methanol, being employed for controlled expression [56]. Enzymes necessitating disulfide bonds for stability or activity should form these bonds correctly within the reducing environment of the yeast cell. Ideally, enzymes should exhibit a codon usage pattern compatible with yeast, optimising translation efficiency. Moreover, enzymes should not induce cytotoxic effects on yeast cells to ensure cell viability and sustained enzyme production. The overexpression of enzymes should be balanced to avoid imposing an excessive metabolic burden on yeast cells, allowing for sustained growth and production.

Early works, as discussed by Ma et al. and Lin et al., highlight the pivotal role of specific structural features essential for catalytic performance in *Pichia pastoris* [57,58]. However, our understanding of comparative homology with other depolymerising enzymes, such as lipase, laccase, and esterase, remains limited. The investigation into the correlation between homology and enzyme properties is still in its nascent stages. As an increasing number of depolymerising enzymes are being reported, homology modelling emerges as an indispensable approach for elucidating the bio-mechanisms underlying the superior degradative capabilities of these enzymes.

Homological analysis of cutinase-like enzymes has revealed a conserved G-Y-S-Q-G domain containing a catalytic S-D-H triad [59], and disulfide bonds in the enzyme contribute to the thermodynamic stability and the kinetic stability of cutinases [60]. The cutinase, which prefers medium- to long-chain substrates, often exhibits depolymerising ability [61]. This property of cutinase has been reported to be associated with different structural features of active enzymatic sites, such as a mutant change on the small helical flap [62] and the presence of a deep continuous groove extending across the active site (in comparison with another cutinase carrying a shallow and interrupted groove at active sites that favours short-chain substrates) [60]. More accessible space at the active site of the enzyme is assumed to facilitate the catalytic activity. As reported, the presence of an extended groove near the catalytic triad (Ser-Asp-His) is important for a better accommodation of polymeric substrates [63], and the enlarging active site was further found to enhance fungal cutinases’ activity for recognising and fitting towards polymer chains like polyethylene terephthalate (PET) and polyamide 6,6 (PA 6,6) fibres [64]. Also, the hydrophobicity of the Ser-Asp-His catalytic triad could determine the affinity ability to amphiphilic long-chain substrates such as PET [65].

The primary focus remains on the continual enhancement of enzymatic stability and catalytic efficiency [66]. Recent insights into optimising protein structures to improve plastic biodegradation performance are highlighted in a comprehensive review [67]. Many polymer-degrading enzymes require high temperatures to be effective, especially for applications involving PET, which has a high glass transition temperature. *Pichia pastoris* has been used to express engineered enzymes with improved thermostability, allowing the enzymes to maintain activity under industrially relevant conditions.

Plastic surfaces are hydrophobic, which can limit enzyme access. To address this, enzymes can be engineered to have enhanced surface-binding properties. Pichia is used to express such modified enzymes; however, the modifications do not compromise enzyme activity or stability.

One illustrative example involves the cutinase from *Aspergillus oryzae*, where the introduction of additional mutations, forming a salt bridge network in its structure, led to a notable increase in enzyme stability of 6 °C. However, this optimisation did not correspondingly enhance catalytic activity [63]. Challenges in enzyme engineering arise from specific characteristics, such as the disparity between the thermo-unfolding temperature of the active site and that of the enzyme’s overall structure, as elucidated in the works of Sulaiman et al. and Shirke et al. [63,65].

## 4. The Influence of Post-Translational Modification During the Overexpression of Depolymerising Enzymes

Enzymes must exhibit specificity for the target substrate, such as plastic polymers, and demonstrate high catalytic efficiency to ensure effective depolymerisation. Compatibility with yeast post-translational modification machinery is crucial for enzymes to undergo necessary modifications, which can significantly impact enzyme stability and activity. Hyper-glycosylation of heterologous proteins [68] is one of the post-translational modifications resulting in changes in the stability and activity of expressed enzymes [69,70]. Sometimes, non-glycosylated variants may be preferable for certain applications, requiring the use of strains engineered for minimal or no glycosylation.

The efficiency of target protein secretion in *P. pastoris* can be also influenced by the different glycosylation states of the protein variants [7,71]. Hyper-glycosylation in *P. pastoris* expression has been particularly postulated to be responsible for influencing the molecular mass, isoelectric point, and pH range of the expressed protein [40,72,73], and even the biodegradation activity [41,44]. The impact of glycosylation on cutinases has been investigated, with early studies determining the presence or absence of N- or O-glycosylation sites prior to fusion [74].

Tammer et al. reported that a cutinase gene deduced from *Aspergillus niger* carries 33 sites for O-glycosylation, and its expression by *P. pastoris* generated a larger target (40 kDa) than expected (29 kDa, taking into account the His-tag), sustaining hydrolytic activity against ester polymers [40]. Glycosylation-site knock-out mutants of a cutinase from *Thermobifida cellulosilytica* showed no difference in the protein expression level but higher hydrolysing activity against poly (butylene succinate) compared with the wildtype protein [41].

Li et al. have reported that a PET-degrading enzyme variant, CtPLDM from *Caldimonas taiwanensis*, was expressed in the industrial strain *P. pastoris* [75]. However, the enzyme initially exhibited inactivity towards PET, contrasting with active expression in *E. coli*. Structural analysis revealed that N-glycosylation at residue N181 restrained the flexibility of a substrate-binding tryptophan, impairing enzyme activity [75]. The glycosylation site restricted the conformational adjustments needed for PET degradation. Another N181A variant was engineered to eliminate the problematic glycosylation, restoring PET hydrolytic activity. Further rational design and molecular engineering expanded the substrate-binding tunnel (F235L), enhancing enzyme performance [75]. This variant cutinase was identified as a promising candidate for additional improvements, such as enhancing thermostability, which is critical for industrial applications.

Another case study by Gamerith et al. revealed a distinct impact of glycosylation on the degradation ability of *Thermobifida cellulosilytica* cutinase 1 (Thc_Cut1) and its glycosylation mutants, Thc_Cut1_koAsn and Thc_Cut1_koST, which were expressed in *P. pastoris*. Thc_Cut1_koST, for instance, was significantly more active on poly (butylene succinate) (PBS) compared to its native form. All Thc_Cut1 and its mutants effectively hydrolysed PET, PBS, and PHBV, with better efficiency on PBS [41]. Thc_Cut1_koST exhibited up to 92% weight loss in PBS films in 96 h, indicating significant potential for biodegradable polyester recycling [41]. Interestingly, the glycosylation site knock-out mutant showed no significant impact on total protein yield, and the removal of glycosylation sites did not reduce performance on PET and, in fact, enhanced activity on PBS [41]. This structural modification implies that glycosylation effects depend on the specific substrate and desired enzyme characteristics.

Differently, Shirke et al. explored leaf and branch compost cutinase (LCC) from bacterial origin and used glycosylation to improve thermal stability and reduce aggregation in the *P. pastoris* expression system [44]. It was found that glycosylation of LCC (LCC-G) increased the temperature tolerance by 10 °C and slowed aggregation, crucial for PET hydrolysis at temperatures above PET’s glass transition temperature, making it a robust candidate for practical recycling applications [44]. In this study [44], the glycosylated cutinase showed an enhanced kinetic stability above the glass transition temperature of PET to achieve better efficiency on PET degradation compared to that of the natively non-glycosylated cutinase. Therefore, investigation of the influence of glycosylation is necessary in each specific case.

## 5. The Impact of Vector Construction on Functional Overexpression

Selecting the appropriate vector for functional overexpression is a critical decision in the pursuit of high-purity depolymerising enzyme expression. The vectors pPICZ and pGAPZ were popularly used to express depolymerising enzymes with high purity in *P. pastoris* [25,76]. The codon optimisation of the target enzyme sequence is crucial for vector construction to enhance protein secretion, stability, and biofunction [77,78,79,80].

Equally influential is the decision regarding signal sequence addition or the engineering of the N-terminal peptide, which significantly impacts the expression level of the foreign protein. Examples are the utilisation of the *Saccharomyces cerevisiae* α-factor signal sequence [23] and other native propeptide signal sequences [78,79]. Signal peptides guide enzymes to the secretory pathway, facilitating extracellular expression [23]. Signal peptides are often optimised to improve enzyme secretion into the culture medium, which simplifies downstream processing. These choices play a pivotal role in dictating the success of the overexpression strategy, offering a nuanced approach to tailoring the outcome based on specific biological and functional requirements [79]. Therefore, an effective secretion signal is necessary to ensure the proper trafficking of the enzyme to the extracellular space, where plastic degradation typically occurs.

The AOX1 promoter, which is inducible by methanol, is often used for high-level expression of plastic-degrading enzymes. However, constitutive promoters like GAP are also explored to avoid the use of methanol, especially when continuous enzyme production is needed.

## 6. Other Factors Influencing Heterogeneously Produced Depolymerising Enzymes

The activity of expressed enzymes could be significantly reduced or lost after filtration, and enzyme purification is a key step to obtaining the target enzyme. Yang et al. demonstrated that the acetyl xylan esterase (AXE, one of the fungal enzymes required for degrading hemicelluloses) exhibited 0.68% of the total initial activity after the purification process but a 26.1-fold increase in specific activity compared with the crude culture filtrate [34].

Vectors constructed with the (His)6-tag are considered a way to achieve easy purification and detection of recombinant protein [81,82]. A redox-responsive cutinase gene (Mfcut1) fused to the (His)6-tag was firstly expressed at high levels in *P. pastoris* using the vector pPIC9K, and the results demonstrated that the recombinant expression of a cutinase could be induced by cutin monomer presence or glucose depletion [83]. Other His-tagged cutinases (e.g., a cutinase from *Aspergillus niger* [84]; a cutinase from the ascomycetous plant pathogen *Sirococcous conigenus* [74]) were subsequently reported in *P. pastoris* expression, and the recombinant enzymes showed activity over a broad range of pHs with maximal activity. On the other hand, a study on a *Fusarium solani* cutinase [85] reported that the addition of (His)6 tag negatively affected a cellular process for proper synthesis, folding, and secretion of cutinase, associated with the generation of two secreted proteins in different molecular weights in the expression cells with the His tag, assuming that other factors of post-translational modification, particularly glycosylation, would determine the activities of expressed proteins.

The high yield and purity of enzymes through a heterogenous expression system does not assure an expect catalytic ability regarding the activity per unit, since enzyme application in high density may cause a negative influence. A *Fusarium verticillioides* cutinase expressed by *P. pastoris* was reported to form numerous tuberculate or warty protrusions on a treated surface when being used in a bioreactor for hydrolysing rice straw, leading to repressed enzymatic activity [86].

Additionally, many metal ions and chemicals play various roles in the stability and depolymerising activity of overexpressed enzymes. The activity of a cloned *Fusarium solani* cutinase was enhanced by K+ and Na+ and inhibited by Zn^2+^, Fe^2+^, Mn^2+^, and Co^2+^ [42]. The activity of overexpressed lipases from *Fusarium solani* was enhanced by Ca^2+^ [23,87], and the structural modelling reveal that the most probable Ca^2+^ binding site is not inside the active site but rather located in a surface loop participating in the hydrophobic interface with the substrate [87]. Another researcher demonstrated that the catalysing efficacy of an expressed lipase from *Aspergillus oryzae* was limited by Zn^2+^ and Cu^2+^ [26]. EDTA and β-mercaptoethanol exerted a significant inhibitory effect by breaking the disulfide bonds, indicating the biofunctional importance of a serine residue in the enzymatic activity [42,87]. Chemicals like Tween-20 showed inhibitory effects on the enzymatic activity, indicating that the cloned enzyme contains hydrophobic groups at its active site [42].

## 7. Strategies Incorporated into Depolymerising Enzyme Overexpression

**Figure 2:**
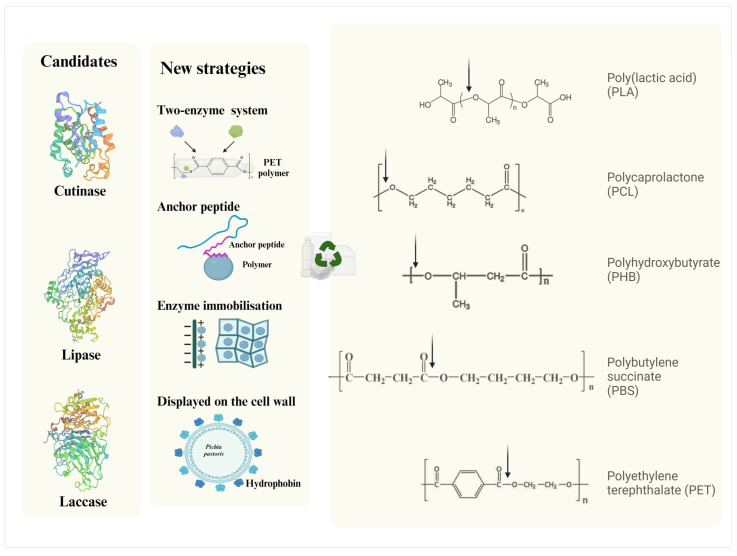
Overview of the heterogenous expression of depolymerising enzymes in the *P. pastoris* system (generated by BioRender).

New strategies for enhancing the overexpression of depolymerising enzymes have been explored (Figure 2), with a focus on well-known enzymes such as PET hydrolases (PETases), which are involved in the degradation of PET. PET hydrolases (PETases) are a crucial class of enzymes that hydrolyse the ester bonds in polyethylene terephthalate (PET), a plastic widely used in bottles, clothing fibres, and food containers. The enzymatic breakdown of PET produces its monomeric components, primarily terephthalic acid and ethylene glycol, which can be recycled into new PET products or repurposed for other uses. The PETase enzyme from *Ideonella sakaiensis* [88,89] gained significant attention after it was discovered for its ability to degrade PET under mild conditions. Researchers have since worked on enhancing the enzyme’s activity and stability to make it more suitable for industrial applications. For example, variants have been engineered to exhibit improved thermal stability, allowing the enzyme to operate effectively at higher temperatures, which increases the plastic’s amorphous regions and facilitates faster hydrolysis [90,91]. Efforts also focus on increasing the enzyme’s catalytic efficiency by modifying its active site and substrate-binding regions.

Expression of dual enzymes has been designated to improve thermal stability [92], with an increase in catalytic efficiency [93,94], to minimise any pre-treatment step for polymer biodegradation [95]. The two-enzyme PETase/MHETase system for PET depolymerisation is a first example, using *E. coli* as the expression host [67,96]. A chimeric lipase–cutinase was successfully overexpressed in *P. pastoris* and exhibited lipase and cutinase activities 127% and 210% higher than their parent enzymes [97], contributing an improved effect of degrading vinyl acetate (PVAC) by the synergistic action of the moieties [97]. Its PCL-degrading ability was subsequently determined, and the weight loss in PCL films with the fusion protein treatment was 14.35, 12.77, and 6.67 times higher than that achieved with lipase and cutinase alone or with a mixture of lipase and cutinase, respectively [98]. The use of an anchor peptide (adhesion promoter) with a target enzyme has been used for the immobilisation of functional protein to different polymer surfaces [99,100,101]. The introduction of material-binding peptide was reported to accelerate the degradation efficiency of the secreted enzyme against polymer nanoparticles [102] and polymers in suspension [103], indicating a bio-strategy of treating waste water containing microplastics.

Enzyme immobilisation on the cell surface of the expression host also showed a positive impact on enhancing the enzymatic activity [104,105]. A lipase B from *Candida antarctica* was reported to be expressed and displayed on the surface of modified *P. pastoris* cells, which co-expressed a hydrophobin responsible for structural and hydrophobic changes in the *P. pastoris* cell surface, and the lipase activity was enhanced on the cell surface with glycerol barrier removal in an anchoring form, causing substrates to easily access the lipase active site [106]. Recently, Chen et al. [80] established a whole-cell biocatalyst model by displaying PETase on the surface of *P. pastoris* cells with significantly enhanced catalytic efficiency, enabling the degradation of commercial PET bottles. This technology can easily produce enzymes from *P. pastoris* (as the expression host) and make the secreted enzyme anchor on the surface of the same *P. pastoris* cell (as the support cell) to avoid the extra process of enzyme separation and purification [107]. The continuing turnover of the anchored enzyme further contributes to a better reusability of the biocatalysis through simply optimising the culture conditions [80], a key to biodegradation engineering for stabilising the catalytic rate in a streamlined process.

## 8. Applications and Industrial Relevance

It is commonly recognised that *P. pastoris* secreting proteins into the culture medium simplifies downstream processing and purification. Its ability to grow on simple, defined media enhances cost-effectiveness, distinguishing it from expression systems requiring more complex formulations. Utilising methanol as a carbon source for induction, *P. pastoris* offers an inducible system that allows for precise control over the timing and level of protein expression, providing a switchable expression system independent of nutrient depletion. The yeast also provides a favourable environment for proper protein folding and secretion, facilitated by its secretion machinery that corrects protein folding, often eliminating the need for extensive refolding during purification. Therefore, *P. pastoris* is well suited for large-scale fermentation, making it suitable for industrial-scale enzyme production, thanks to its scalability and robust fermentation characteristics [24].

Enzymatic depolymerisation requires highly effective enzymes on a large scale and are in high demand. This section highlights the *pichia*-overexpression of polyesterase, which demonstrated activity against some bioplastics, showcasing their potential for sustainable application and the technological possibilities when moving to the scale-up stage. In this context, we focus on two commonly used biodegradable and recyclable polyesters—poly(lactic acid) (PLA) and polycaprolactone (PCL)—as promising alternatives to conventional plastics [27].

Poly(lactic acid) (PLA) is a compostable thermoplastic polyester that can be derived from renewable resources like corn, sugarcane, or cassava. Nowadays, demand for it has grown, especially in food- and beverage-packing materials. As the use of biodegradable plastics like polylactic acid (PLA) continues to grow [108], there is an increasing need for efficient recycling and disposal methods. Enzymatic degradation represents a potential solution in current scientific investigation. A recent study evaluated the suitability of cutinases derived from *Aspergillus nidulans* and expressed in *P. pastoris* to recover lactic acid and create higher-value products. They found that one enzyme, ANCUT1, produced a significant amount of L-lactic acid—higher than that produced by proteinase K, a known PLA-degrading enzyme [109]. Comparatively, Carbios has reported significantly higher enzymatic efficiency, with yields 2.23 times greater than those achieved with ANCUT1 (0.91 mmol lactic acid/g enzyme/h^−1^) [109,110]. This discrepancy underscores the need for optimisation, leading to efforts focused on factors such as PLA particle size/form, reaction temperature, pH, and enzyme concentration [109]. In a related approach, the cutinase secreted by *Fusarium solani* (FsC) was heterologously produced in high yields, and its hydrolytic efficiency was evaluated on PLA polymers with varying stereochemistry, crystallinity, and degrees of polymerisation [111]. Under the experimental conditions, FsC exhibited enantioselectivity, demonstrating optimal activity on poly-D,L-lactic acid (PDLLA), while showing no hydrolytic activity on poly-L-lactic acid (PLLA) [111]. The hydrolysis of PDLLA was further optimised using response surface methodology, resulting in an 88% hydrolysis rate within 10 h under the optimised conditions [111].

The findings suggest that PLA particle size plays a critical role in balancing enzyme–substrate contact and efficient product release. Similarly, enzyme concentration was observed to impact yield, as excessively high enzyme levels may interfere with the mobility required for effective degradation reactions. These insights provide a foundation for refining enzymatic processes to improve PLA-recycling efficiency.

Polycaprolactone (PCL) is a biodegradable polyester with a low melting point that is widely used in applications such as biomedical devices, drug-delivery systems, and packaging materials. In terms of recycling and sustainability, PCL is also considered more eco-friendly than conventional petroleum-based plastics. However, its degradation rate in natural environments is relatively slow compared to other bioplastics. Efforts are being made to improve its cycling through enzymatic and chemical recycling methods. Enzymatic depolymerisation, driven by specialised enzymes capable of breaking down polyester bonds, shows promise for enhancing PCL’s recyclability by converting it back into its monomer form, which can then be repolymerised to create new products. Current research is focused on optimising these biotechnological processes.

Oh et al. successfully screened for PCL-depolymerising enzymes, identifying a superior variant, CalB-658, through a PCL emulsion agar plate assay [17,18]. CalB-658, a modified lipase B from *Candida antarctica*, demonstrated significantly enhanced activity compared to the wildtype enzyme, depolymerising 97.3% of PCL films over 32 h [17]. Using *P. pastoris* as the recombinant host, CalB-658 was produced extracellularly in large quantities (844.3 mg/L), with a lipase activity of 13,753.3 U/L [17]. This production system leverages the strong, methanol-inducible AOX1 promoter, simplifying the purification process due to low levels of endogenous secreted proteins. CalB-658 showed a significant improvement in converting PCL to 6-hydroxyhexanoic acid (6-HHA), yielding 5.0-fold more 6-HHA than the wildtype enzyme [17]. This study further explored the conversion of 6-HHA into value-added biochemicals, such as succinic acid and polyhydroxyalkanoate (PHA), using metabolically engineered strains of *E. coli*. Overall, the use of *Pichia pastoris* as a host for expressing plastic- and bioplastic-degrading enzymes presents a versatile and scalable approach for industrial applications [17].

The current lab bioreactor testing shows that CalB-658 exhibits plastic degradation (60 mg) over 32 h, indicating a time-dependent activity that may limit its immediate real-world application. However, the enzyme could be further engineered to accelerate the degradation rate. Engineered enzymes could then be employed in large-scale plastic waste recycling by optimising enzyme concentration and reaction time in bioreactors to break down plastics like PCL into reusable monomers. For environmental cleanup, enzymes could also be immobilised or used in biofilms to enhance plastic degradation in contaminated sites.

Alternatively, one promising approach is the development of enzyme cocktails, which combine multiple enzymes to degrade a wider range of plastic polymers simultaneously. These cocktails could be produced in co-cultures or through engineered *Pichia* strains capable of co-expressing complementary enzymes, making them more effective at tackling mixed or multilayered plastic waste. Such innovations hold potential for large-scale bioremediation efforts, offering a proactive solution to plastic pollution in both terrestrial and aquatic ecosystems. The industrial relevance of these enzymes is underscored by their potential to transform how we handle plastic waste, providing eco-friendly alternatives that align with global environmental goals.

## 9. Future Directions and Research Focus

The complexity of plastic waste requires a multifaceted approach to biodegradation. To address this, *Pichia pastoris* strains are being engineered to co-express multiple enzymes capable of working in tandem to degrade plastics more efficiently. For instance, PET, one of the most common plastics, can be broken down into simpler monomers through a series of enzymatic reactions [112]. By co-expressing enzymes such as PETases [113], which initiate the breakdown of PET into smaller oligomers, and MHETases, which further hydrolyse mono(2-hydroxyethyl) terephthalate (MHET) into terephthalic acid and ethylene glycol, a sequential and more effective degradation process can be achieved. Additional enzymes like cutinases can complement this process by enhancing the breakdown of polyesters and mixed plastic materials [91]. The challenge lies in ensuring these enzymes are expressed at appropriate levels, remain stable, and function efficiently within the same biological system. Optimising expression cassettes and regulatory elements to balance enzyme production, minimise metabolic burden, and prevent potential interference between enzymes is an ongoing area of research [114]. Moreover, the development of *Pichia* strains that can efficiently secrete these enzymes into the extracellular environment is essential for practical applications in bioremediation and recycling processes.

By designing *Pichia* strains with synthetic gene circuits and optimised metabolic pathways, researchers can fine-tune the production and secretion of plastic-degrading enzymes. These advancements include the use of CRISPR-based genome editing [115] to insert or delete genes with high precision, as well as the implementation of synthetic promoters and transcriptional regulators to control gene expression. Additionally, synthetic biology enables the integration of biosensors into *Pichia* cells, allowing for real-time monitoring of enzyme production and cellular health [116]. These biosensors can be designed to respond to specific signals, such as the presence of plastic degradation products or changes in pH, providing feedback that can be used to adjust fermentation conditions dynamically. Furthermore, efforts are being made to incorporate pathway engineering strategies that reroute cellular resources towards enzyme synthesis, thereby maximising productivity. The integration of synthetic metabolic pathways can also support the co-production of valuable by-products, adding economic value to the biodegradation process.

A major focus in the development of *Pichia* as a production host for plastic-degrading enzymes is the sustainability of the bioprocesses involved. Traditionally, *Pichia pastoris* uses methanol as an inducer for the AOX1 promoter, but methanol is both flammable and derived from fossil fuels, posing safety and environmental concerns [117]. To address this, researchers are developing methanol-free fermentation systems that employ alternative promoters, such as the constitutive GAP promoter or novel inducers that are safer and more sustainable [118]. These methanol-free systems not only reduce the environmental impact but also simplify the fermentation process, making it more cost-effective and suitable for large-scale applications. Additionally, efforts are being made to use renewable and low-cost feedstocks, such as agricultural residues, waste glycerol from biodiesel production, or lignocellulosic biomass, as carbon sources for *Pichia* growth and enzyme production [117]. This approach leverages existing waste streams, turning them into valuable inputs for enzyme synthesis and further contributing to the circular economy. Process strategies, such as continuous fermentation and the use of high-cell-density cultures, are also being explored to increase productivity and reduce resource consumption [117].

## 10. Summary

Plastics depolymerisation poses a challenge due to the demanding redox potential requirements, often exceeding those exhibited by most oxidoreductases [119]. This review advocates for the heterogenous expression of depolymerising enzymes in the *P. pastoris* system, which has demonstrated success in many in vitro cases. Numerous enzymes expressed in *P. pastoris* exhibit potential for a complete plastic depolymerisation in vitro. Table 1 summarises the key studies.

The current focus is on sustaining a bioanalysing reaction and the emergence of a novel biocatalysis system [79], showing a promising role of the *P. pastoris* fermentation system in developing a biocatalysing reactor that closely mirrors real-world conditions. The supportive outcomes open up prospects for advancing plastic depolymerisation technologies.

The expression of plastic- and bioplastic-degrading enzymes in *Pichia pastoris* represents a significant advancement in the quest for sustainable waste management and environmentally friendly recycling methods. By leveraging Pichia’s ability to produce high yields of active enzymes, researchers are developing bio-based solutions that could revolutionise how we address plastic waste. Enzymes such as PETases, cutinases, and polyesterases are being engineered and optimised for enhanced activity, stability, and efficiency under various environmental and industrial conditions. These efforts are making the idea of enzymatic recycling more feasible and scalable.

Despite the progress, the efficient degradation of highly crystalline plastics, enzyme stability under harsh industrial conditions, and the economic feasibility of large-scale bioprocesses are the main challenges requiring further research. Additionally, the optimisation of fermentation processes to reduce costs, improve yields, and enhance the sustainability of enzyme production is crucial. Innovations in protein engineering, such as developing multi-enzyme systems and synthetic biology approaches, are driving this field forward, while methanol-free fermentation and the use of renewable feedstocks are aligning enzyme production with eco-friendly practices. The continued collaboration between scientists, industry partners, and policymakers will be essential to realise these promising solutions on a global scale.

## Figures and Tables

**Table 1:** Key studies on heterogenous expression of depolymerising enzymes in *Pichia pastoris*.

Enzyme	Polimer	Enzyme Mass	Activity/Yield	pH/Temp (°C)	Degrading Efficacy	Ref.
*Aspergillus niger* lipase (without the signal peptide sequence)	PLA PCL	The native peptides at 35 and 37 kDa (the bigger molecular mass due to glycosylation); the purified recombinant at higher sizes (43 and 45 kDa) due to the His-tag in the protein	Vmax of 32.21 μmol/min/mg and Km of 3.83 mM using olive oil as substrate	pH of 4.0 to 10.0, temp of 37 to 50 °C, optimal pH and temperature 7.0 and 37 °C	Ten mg/mL of each plastic was degraded by the lipases, and the degradation % was calculated by measuring the decrease in turbidity of the emulsions at 580 nm before and after addition of the enzyme), with 87 and 83% loss of PLA 5000, 75 and 84% loss of PLA 10,000, and 78 and 31% loss of PCL 10,000 at 30 °C in 72 h.	[27]
*Candida Antarctica* lipase B	PCL	34 kDa	At a concentration of 844.3 mg/L with a lipase activity of 13.75 U/mL, using ρ-NPP (C6) as substrate	Optimal activity at 50 °C and pH 7.0	A lipase mutant (CalB-658) degraded 97.3% of 60 mg PCL film at 32 h, and the production of the monomer of PCL (6-HHA) was further improved in a bioreactor at 50 °C.	[17,18]
*Thermobifida* sp. polyhydroxyalkanote depolymerase	PHB	Two glycosylated forms at 61 and 70 kDa	Vmax of 3.63 ± 0.16 μmol/min/mg protein and Km of 0.79 ± 0.12 mM using ρ-NPB (C10) as substrate	Optimal activity at 50–55 °C and pH 7.0–8.0	The degradation rate of PHB was linear for the first 400 min, estimated at 130 ng cm^−2^ h^−1^, and declined thereafter.	[31]
*A. oryzae* cutinase; *F. solani* cutinase	PCL	N/A	*F. solani* cutinase: Km of 1.50 µM using ρ-NPH (C12) as substrate *A. oryzae* cutinase: Km of 4.96 µM using ρ-NPA (C8) as substrate	High reactivity at 40 °C and pH 8.0 for both enzymes	87% of PCL weight loss (mg/cm^2^) in the presence of *A. oryzea* cuitnase and 30% by *F. solani* cutinase within 6 h	[60]
*F. solani* cutinase	PBS	24 kDa	Km of 1.37 mM using ρ-NPB as substrate	pH of 4.0 to 10.0, temp of 20 to 50 °C, optimal pH and temperature were 8.0 and 50 °C [6]	100% weight loss in PBS films (30 × 10 × 0.1 mm) after 6 h at 50 °C [6]; 100% weight loss in PBS films (30 × 10 × 0.5 mm) after 26 h incubation at 37 or 45 °C [7]	[37,42]
*F. solani* cutinase; *A. fumigatus* cutinase	PCL	Similar size at 20 kDa	Using ρ-NPB as substrate, 4370.5 U/g of *F. solani* cutinase and 797.2 U/g of *A. fumigatus*	*F. solani* cutinase pH 7.5 and 40 °C; *A. fumigatus* cutinase pH 8.0 and 60 °C	At 40 °C, *A. fumigatus* cutinase completely degraded the PCL films after 6 h, while *F. solani* degraded it by 44.3% after 12 h.	[43]
*C. antarctica* lipase, *F. solani* cutinase	PCL	N/A	45 U/mL lipase or 45 U/mL cutinase using ρ-NPB as substrate	Lipase at 45 °C and pH 7.2 or cutinase at 37 °C and pH 7.2	87.56% (lipase) and 80.8% (cutinase) weight loss in PCL films after 72 h of incubation	[38]
*Thermobifida cellulosilytica* cutinase	PBS PET	38 kDa (wildtype) 29.4 kDa (deglycosylated mutants)	100–210 U/mg using ρ-nitrophenyl butyrate (ρ-NPB) as soluble substrate	65 °C and pH 8.0	After 96 h of incubation, ~24% degradation in initial PET powder to soluble TPA was seen in both wildtype and mutant enzymes; ~24 and 48% degradation in initial PBS powder to soluble released products by wildtype and mutants, respectively.	[41]
Cutinase (leaf and branch compost)	PET	N/A	200–300 mg/L of glycosylated LCC produced by *P. pastoris*	70 °C pH 8	∼95% weight loss in PET film (1 cm × 1 cm and 250 μm thick) after 48 h incubation	[44]
*Glomerella cingulata* cutinase	PET	25 kDa	k_cat_/K_m_ of 7.7 ± 0.7 mM^−1^ s^−1^ using ρ-NPC (C8) as substrate	25 °C pH 8	PET film surface was peeled, pitted, and corroded under a scanning electron microscope after 24 h incubation.	[61]
Lipase–cutinase fusion enzyme (*Thermomyces lanuginosus and Thielavia terrestris*)	PCL	50 kDa	188.1 mg/L	40 °C pH 8	The weight loss in PCL films reached 91.95% after 6 h degradation by Lip–Cut.	[98]

Note: All listed enzymes are heterogeneously expressed in *P. pastoris* using vector pPICZαA. The degradation outcomes were achieved in vitro using purified enzymes from the recombinant expression.

## Data Availability

Not applicable.

## Abbreviations

PLA	polylactic acid
PCL	polycaprolactone
PHB	poly-[(R)-3-hydroxybutyrate]
PBS	poly (butylene succinate)
ρ-NPP	ρ-nitrophenyl palmitate
ρ-NPB	ρ-nitrophenyl butyrate
ρ-NPH	ρ-nitrophenylhexanoate
ρ-NPA	ρ-nitrophenylacetate
6-HHA	6-hydroxyhexanoic acid
ρ-NPC	ρ-nitrophenylcaprylate
